# Clinical outcomes of a novel flowable calcium silicate cement in equine endodontic treatment

**DOI:** 10.1002/evj.70299

**Published:** 2026-08-05

**Authors:** Kirsten Jackson, Josephine Faulkner, Carsten Staszyk, William Ha, Ebony Quintrell, Erin Kelty

**Affiliations:** ^1^ Dental Vet Veterinary Equine Dentistry Perth Western Australia Australia; ^2^ School of Veterinary Medicine Murdoch University Murdoch Western Australia Australia; ^3^ Faculty of Veterinary Medicine Institute of Veterinary‐Anatomy, ‐Histology and ‐Embryology, Justus‐Liebig‐University Giessen Giessen Germany; ^4^ Head of Endodontics, Sydney Dental School University of Sydney New South Wales Australia; ^5^ School of Population and Global Health School of Human Sciences, University of Western Australia Perth Western Australia Australia

**Keywords:** apical periodontitis, calcium hydroxide, eCSC, horse, MTA, orthograde treatment

## Abstract

**Background:**

Orthograde endodontic treatment of equine cheek teeth is performed infrequently with variable success rates of obturation with calcium hydroxide paste (CHP). A novel flowable calcium silicate cement (eCSC) formulated for use as an obturation material in equine teeth has demonstrated acceptable canal filling volumes and evidence of dentinal tubule penetration. However, clinical outcomes of eCSC‐obturated equine teeth have not been reported.

**Objectives:**

To report the clinical outcomes of orthograde endodontic treatment using eCSC.

**Study Design:**

Retrospective case series.

**Methods:**

Horses that underwent orthograde endodontic treatment using eCSC at a single referral centre between October 2020 and March 2026 and had ≥12 months follow‐up were included in the study.

**Results:**

Seven horses, with endodontic treatments on six cheek teeth and one incisor were included. The success rate of eCSC was 6 of 7 cases, with up to 5.5 years of follow‐up. One eCSC case demonstrated healing of a draining tract to the paranasal sinus on CT and histologically showed no evidence of periapical infection or inflammation 16 months after treatment. The singular failed eCSC‐treated tooth demonstrated incomplete cleaning and obturation of one pulp canal on histological examination.

**Main Limitations:**

Small case numbers, heterogeneous follow‐up, and a single operator limit the generalisability of these findings.

**Conclusions:**

Orthograde endodontic treatment of equine teeth using eCSC can result in long‐term clinical success. CT and histopathological findings available for one case support biological healing in successfully treated eCSC cases. A prospective, multi‐centre study with standardised follow‐up is warranted to establish the predictability and indications of eCSC as an obturation material for equine endodontics.

## 1 INTRODUCTION

Apical infections of equine cheek teeth carry serious clinical consequences, including odontogenic sinusitis, draining tracts, facial swellings, and pain‐related behavioural changes.^1^, ^2^, ^3^ While extraction has historically been the treatment of choice,^1^ it is not without risk, with reported complication rates for cheek tooth extraction ranging from 13.6% to 80%.^4^, ^5^, ^6^, ^7^ Additionally, the sequelae of tooth loss, including dental overgrowths, diastema formation, and secondary periodontal disease, can have lifelong implications for the horse.^3^, ^8^

Orthograde endodontic therapy offers a tooth‐preserving alternative, yet its adoption in equine practice remains limited by technical challenges and published success rates range widely from 44% to 85%.^3^, ^9^, ^10^ Conventional treatment protocols in equine practice typically employ calcium hydroxide paste (CHP) as a canal filling material, supplemented by a calcium hydroxide cement layer and a resin composite occlusal restoration.^10^ The occlusal resin seal is important because the CHP is soluble, meaning that, when exposed, the material will leave the canal, leaving a substantial space for bacterial reinfection.^11^, ^12^, ^13^, ^14^ For this reason, in human endodontics, CHP is classified as a temporary inter‐appointment dressing rather than a definitive obturation material.^11^, ^12^, ^13^, ^14^ Due to the continued eruption and attrition of the equine dentition, the CHP typically becomes exposed after a number of years. If the occlusal resin seal is still in place but thinned, this can be burred away along with the more occlusal aspects of the CHP filling and resealed to ensure further longevity of the endodontic treatment. If the CHP is exposed, the entire endodontic procedure requires repeating. Conversely, another group of endodontic materials called calcium silicate cements (CSCs), that have only recently been used in equine endodontics,^15^ set firmly within the tooth. Therefore, while the occlusal restoration would still require replacement as it is worn through, the CSC sets firm and still seals the canals, so only the occlusal portion requires replacement.

CSCs, of which mineral trioxide aggregate (MTA) is the best‐known representative, were introduced into human endodontics in 1993^16^ and are now employed for a broad range of indications, including pulp capping, apexification, root perforation repair, and, more recently, full canal obturation.^17^, ^18^, ^19^ CSCs are valued for their excellent biocompatibility, sustained alkaline pH, moisture tolerance, non‐shrinking properties, bacterial sealing capacity, and ability to stimulate dentinogenesis, periapical tissue repair, and mineralisation.^17^, ^20^, ^21^, ^22^, ^23^ As an obturation material, CSC outperforms CHP in apexification and resolution of apical periodontitis, as well as in tooth survival,^24^, ^25^ and CSC obturated canals demonstrate greater resistance to root fracture in human studies.^26^ Until recently, the application of CSCs to equine endodontics has been hindered by the handling characteristics, setting behaviour and expense of available products, as well as by the anatomical complexity and access limitations inherent to equine cheek teeth.^27^, ^28^, ^29^ To address these constraints, a novel flowable CSC formulated for equine use (eCSC) was developed following consultation with specialist endodontists.^15^ This material employs a Portland cement/bismuth oxide base combined with an organic diol compound to produce a syringe‐deliverable consistency suitable for injection into the complex anatomy of equine cheek teeth.

A preceding in vitro study examined 10 extracted equine cheek teeth treated with eCSC or CHP, using microcomputed tomography (micro‐CT) and histology to assess canal filling volume and dentinal adaptation.^15^ Mean volumetric fill with eCSC was comparable to the CHP control. Histological analysis demonstrated penetration of eCSC particles into dentinal tubules and the probable formation of an interfacial mineralised layer; findings consistent with the intratubular mineralisation and bacterial entombment described for MTA in human teeth.^20^, ^30^ These laboratory results support progression to clinical application and the aim of the current study was to report the clinical outcomes of orthograde endodontic treatment using eCSC in horses treated at a single referral centre.

## 2 MATERIALS AND METHODS

### 2.1 Study design and case selection

This anonymised, retrospective study included horses that had undergone orthograde endodontic treatment using eCSC at Dental Vet, Perth, Australia, between October 2020 and March 2026. Endodontic treatment was offered as an alternative to extraction if there were one or more non‐vital pulp exposures and apical infection was found on diagnostic imaging. Endodontic therapy was not offered if there was deemed to be a high risk of fracture, such as grade 3 or higher infundibular caries affecting both infundibula, or if there was substantial loss of clinical crown from a previous dental fracture. Young (<6 years old) or older (>20 years old) horses were not typically offered endodontic treatment due to concerns about the increased width of the pulp canals and risk of fracture in young horses or having difficulty gaining canal access in older horses. An active sinus infection was not seen as a reason to avoid endodontic therapy. At the time of treatment, signed, informed owner consent was obtained for all procedures. Horses were eligible for inclusion in the study if they had a minimum follow‐up of 12 months. Cases with complete dentinal bridges and no apical infection were excluded from the study due to the fundamentally different nature of the disease and to avoid artificially inflating success rates.

Each complete endodontic procedure of an affected tooth, including any necessary subsequent repair of the occlusal sealant, is referred to as a case in this publication.

### 2.2 Endodontic technique

All procedures were performed by a single operator (Kirsten Jackson) with the horses standing and sedated following the orthograde technique described in Jackson et al.^15^ Horses were sedated and placed in stocks on a detomidine‐butorphanol or detomidine‐morphine constant rate infusion titrated to effect, and intravenous fluids were administered throughout the procedure. A rigid dental endoscope (Karl Storz Endoscopy) was used for cheek teeth. The decision to perform the endodontic procedure in a single or split treatment was based on whether the canals could be adequately cleaned and disinfected (paper points and files coming out completely clean) in a single session or if a second treatment session was required. If the canals could not be adequately cleaned within a reasonable sedation time during one treatment period, they were filled with CHP, which was compressed, and a layer of calcium hydroxide cement (Provicol, Voco) was placed as a temporary dressing to allow continued disinfection. At the second treatment, a few weeks later, the CHP and calcium hydroxide cement were completely removed before further cleaning, and the final obturation and restoration were performed.

The protocol for treatment of the tooth involved establishing occlusal access using high‐speed, water‐cooled round and Lindeman burs (iM3). Working length was determined by radiographic confirmation with nickel‐titanium or stainless‐steel endodontic hand files (60–80 mm, iM3), or in later cases, once CT was available, approximate working lengths could be calculated prior to commencing the procedure from measurements on the images. Chemomechanical preparation employed sodium hypochlorite irrigation (2% NaOCl; Milton's solution, Procter & Gamble) delivered via a negative‐pressure system (where sodium hypochlorite was gently injected into one canal while suction was applied to an adjacent, joining canal, drawing the disinfectant through the common pulp chamber while minimising the risk of apical extrusion). This was also supplemented by EDTA file lubricant (iM3) with thorough rinsing in between to prevent degradation of the disinfectants,^31^ and Control Cut microbrush canal scrubbing (iM3) to further clean the canals. Canals were shaped with nickel‐titanium rotary (Rotary File, iM3) and reciprocating files (VetFlex&Preserve, Dentsply Sirona) and dried with suction and paper points.

The eCSC was prepared by mixing 5 g of eCSC powder with 1.5–3 mL of eCSC liquid to form a cohesive, injectable paste. The paste was loaded into a 3 mL Luer‐lock syringe with an 18G blunt drawing up needle attached and gently curved to 90 degrees to allow insertion into the tooth. The needle was inserted as far as possible, and the eCSC was injected as far apically as possible within the canal, with continuous injection as the needle was slowly withdrawn. Adjacent communicating canals were generally also filled via the common pulp chamber as the material was injected. Compaction was performed with Lentulo spiral fillers (iM3) on the Deger Y‐Smart Mini Dental Cordless endodontic handpiece (Foshan Deger Medical Equipment), endodontic H‐files, and dental cotton swabs. Approximately 10 mm of canal space was left unfilled occlusally. The occlusal seal was established by firstly placing a thin layer of bulk‐fill resin composite (Bulk EZ) over the eCSC. The walls were then gently rinsed and scrubbed to remove the eCSC, and etched (37% phosphoric acid, iM3), followed by the application of Scotchbond Universal Adhesive (Solventum), and a resin composite (BulkEZ, Zest Dental Solutions) placed occlusally.

### 2.3 Follow‐up and outcome assessment

Ongoing monitoring was conducted as part of routine clinical practice by the treating clinician, with dental examination and radiographs taken at nine to 12 months after the procedure, then every 1–2 years. Detailed monitoring of the occlusal surface during dental examinations after treatment was deemed critical, as due to the continued eruption and attrition in equine dentition, the eCSC became exposed after approximately 2–3 years in most cases. In the experience of the lead author, in previous CHP cases, once the resin composite seal was worn away and Provicol/CHP was exposed, the entire endodontic procedure generally required repeating. Conversely, for eCSC, once the grey eCSC could be seen under the restorative layer, only the occlusal restoration required replacement.

Radiography was performed with a digital system (iCR3600 (iCRco) between 2016 and 2023, Luna 1013XT DR (iRay technology) between 2023 and 2026). In later cases, CT assessment became available and allowed for more detailed examination and evaluation of the success or failure of the procedure in some cases (Canon Aquilion Exceed LB, Canon ANZ), with a multi‐directional mobile platform (Qalibra).

Histological evaluation was performed on select cases, where possible. The sample material was fixed in neutral‐buffered 10% formalin. From the available CT or micro‐CT datasets, areas of the teeth were identified for further microscopic examination. The teeth were then sectioned along defined cutting lines using a diamond‐tipped band saw (MBS 240/E, Proxxon S.A.). The section planes were first recorded under a stereomicroscope (Leica Investa 3, camera: K3C; Leica Microsystems GmbH), and subsequently, tooth sections selected for microscopic examination were decalcified in EDTA solution (ethylenediamine tetraacetic acid disodium salt dihydrate, Carl Roth GmbH + Co. KG), pH 8, at room temperature for approximately 6 weeks. Subsequently, samples were embedded in paraffin wax using an automatic embedding system (EG1150h, Leica Biosystems Nussloch GmbH). For histological preparations, 7‐μm thick consecutive sections were cut with a slide microtome (RM2255, Leica Microsystems GmbH). The sections were mounted on standard slides (Fischer Scientific GmbH) and were stained with toluidine blue according to a routine staining protocol. Finally, sections were examined by light microscope (Leica DM2500, camera: DMC4500; Leica Microsystems GmbH).

Outcomes were derived based on extrapolation from human dental criteria and equine endodontic criteria which typically include assessing the clinical, radiographic and histological outcomes,^32^, ^33^ and was classified as:

Success: resolution or absence of pre‐treatment clinical signs (including nasal discharge, pain‐related behaviour), persisting occlusal seal of restorative material, and radiographic/CT stability or improvement (reduction/stasis of periapical changes including reduction/resolution of periapical lucency, reduction/resolution of periapical sclerosis, resolution of draining tract),^33^ ± histological confirmation of absence of infection or inflammatory infiltrate.

Failure: persistence or recurrence of clinical signs, oral examination changes (such as non‐vital pulp exposure (NVPE) on adjacent, untreated pulp canals), radiographic/CT deterioration (persistent or new periapical lucency, persistent/new draining tracts),^33^ ± histological confirmation of treatment failure.

## 3 RESULTS

### 3.1 Case demographics

In the study period, 10 horses (five geldings and five mares) ranging in age from 9 to 23 years of age underwent 15 endodontic procedures on cheek teeth or incisors with eCSC. A total of seven cases of endodontic treatments in seven horses had greater than 12 months of follow‐up for outcome assessment and were included in the study (Table S1). Initial presenting signs included NVPE noted on oral examination on affected teeth (*n* = 7), evidence of apical infection on radiography (*n* = 7) and CT (*n* = 1), clinical signs such as quidding (*n* = 1), and odontogenic nasal discharge (*n* = 1; with a second considered to have prodromal signs based on the presence of a fistulous tract visible on CT examination). Of the seven cases, three were completed in a single treatment and four required the treatment to be split into two sessions which were performed 2–4 weeks apart.

### 3.2 Follow‐up and clinical outcomes

Repeat dental examinations and radiographic assessments were performed for all cases, but the frequency and nature of follow‐up examinations varied across cases, reflecting the nature of clinical practice and the progressive availability of advanced imaging (Table S1).

Six of the seven cases were classified as successful, with follow‐up periods ranging from 16 months to over 5 years. Five of the successful cases were cheek teeth and one incisor. All cases showed resolution of clinical signs, including nasal discharge (Case 2) as well as improvement and stability in the appearance of the periapical region on radiographs (Figure 1). CT imaging during the treatment phase and post‐treatment was available for one horse (Case 7), which showed evidence of sequential healing of a small fistulous tract communicating with the sinus (Figure 2).

**FIGURE 1:**
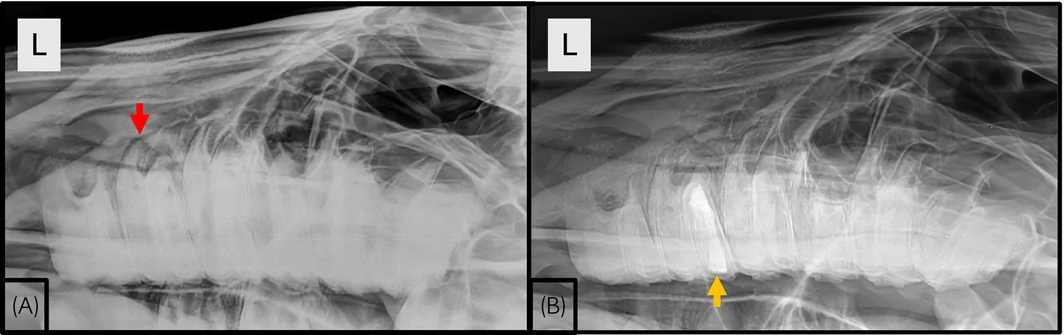
Sequential right dorsal‐left ventral oblique radiographs of the left maxillary cheek teeth arcade of Case 2 before and after endodontic treatment of Triadan 207 with equine calcium silicate cement (eCSC). Pre‐treatment radiograph (A) demonstrating periapical radiolucency of Triadan 207 with marked surrounding sclerosis (red arrow). Follow‐up radiograph at 4 years 10 months (B), showing reduction in the width of the periodontal ligament space, reduced opacity of the alveolar bone and presence of the eCSC obturation material in the region of pulp canals 2 and 4 (yellow arrow).

**FIGURE 2:**
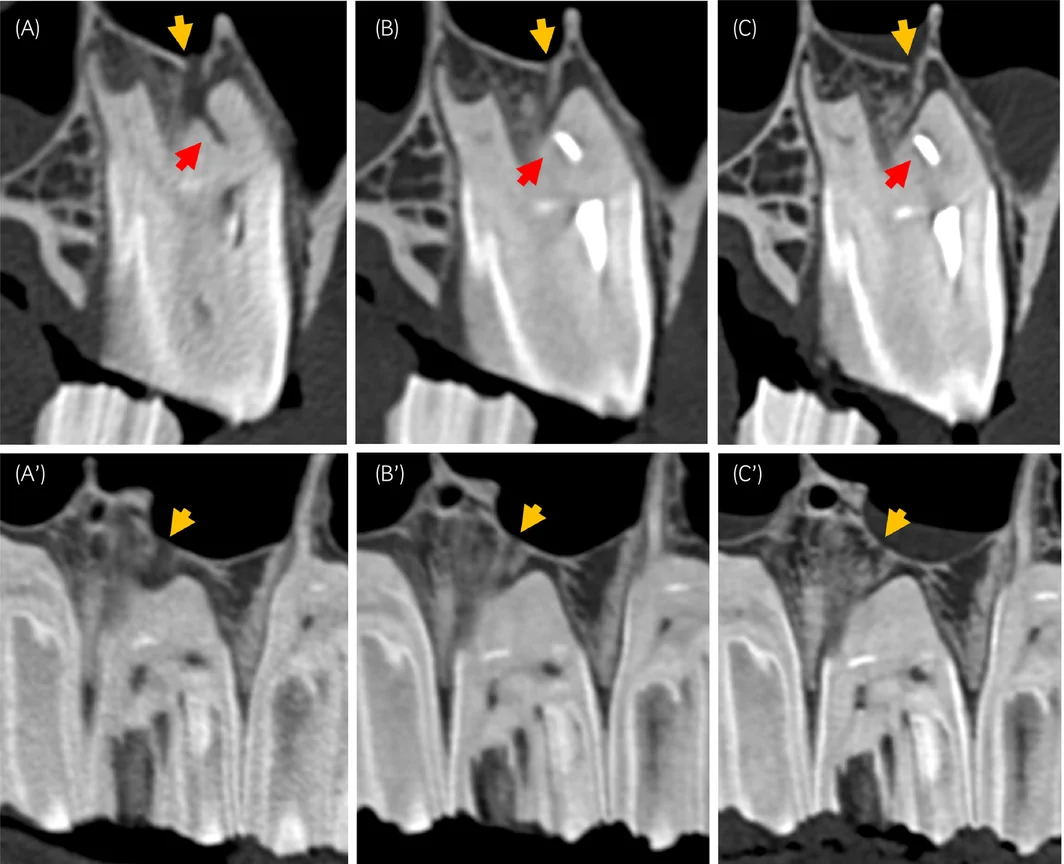
Sequential oblique transverse plane (A–C) and sagittal plane (A′–C′) computed tomographic (CT) images in Case 7, showing the evolution of Triadan 209 following treatment with equine calcium silicate cement (eCSC). CT images during the treatment phase (A and A′) show a fistulous tract to the rostral maxillary sinus (yellow arrow) that communicates with the root canal in the mesiobuccal root (red arrow). Follow‐up CT at 12 months following treatment (B and B′) show narrowing of the fistula with surrounding sclerosis (yellow arrow) and eCSC within the root canal (red arrow). Post‐mortem CT images at 16 months following treatment (C and C′) show narrowing of the periodontal space, further narrowing of the fistulous tract towards the apex of the tooth (yellow arrow) and stable appearance of the eCSC (red arrow). There is fluid in the sinus that represents post‐mortem change.

One eCSC case was classified as a failure (Case 6) due to the adjacent untreated pulp canals becoming open occlusally and the absence of evidence of healing on radiographs. Repeating the endodontic procedure on the remaining pulps was discussed with the owner, but the decision was made to extract the tooth. Concerns later developed regarding the horse's natural immune system capabilities due to consistent extremely high faecal egg counts and development of NVPEs with apical infection in five cheek teeth over a 6‐year period. A subsequent diagnosis of Pituitary Pars Intermedia Dysfunction (PPID or ‘Equine Cushing's disease’) was made.

### 3.3 Histopathological findings

Histological examination was performed on two eCSC cases, with the owners consenting to the teeth being submitted for histological assessment. In Case 6, the tooth was extracted, and in Case 7, the tooth and alveolus were removed from the maxilla following the euthanasia of the animal for reasons unrelated to this study, preserving the surrounding periodontal tissues.

In the failed case (Case 6), sections demonstrated incomplete mechanical debridement with residual necrotic pulp tissue, trapped food material and bacterial contamination in the inadequately treated pulp compartments (pulp horn 2 and the associated distobuccal root canal). A successful filling was present in the pulp horn 4 in the same tooth, featuring adequate obturation and apexification of the associated distopalatal root canal (Figure 3).

**FIGURE 3:**
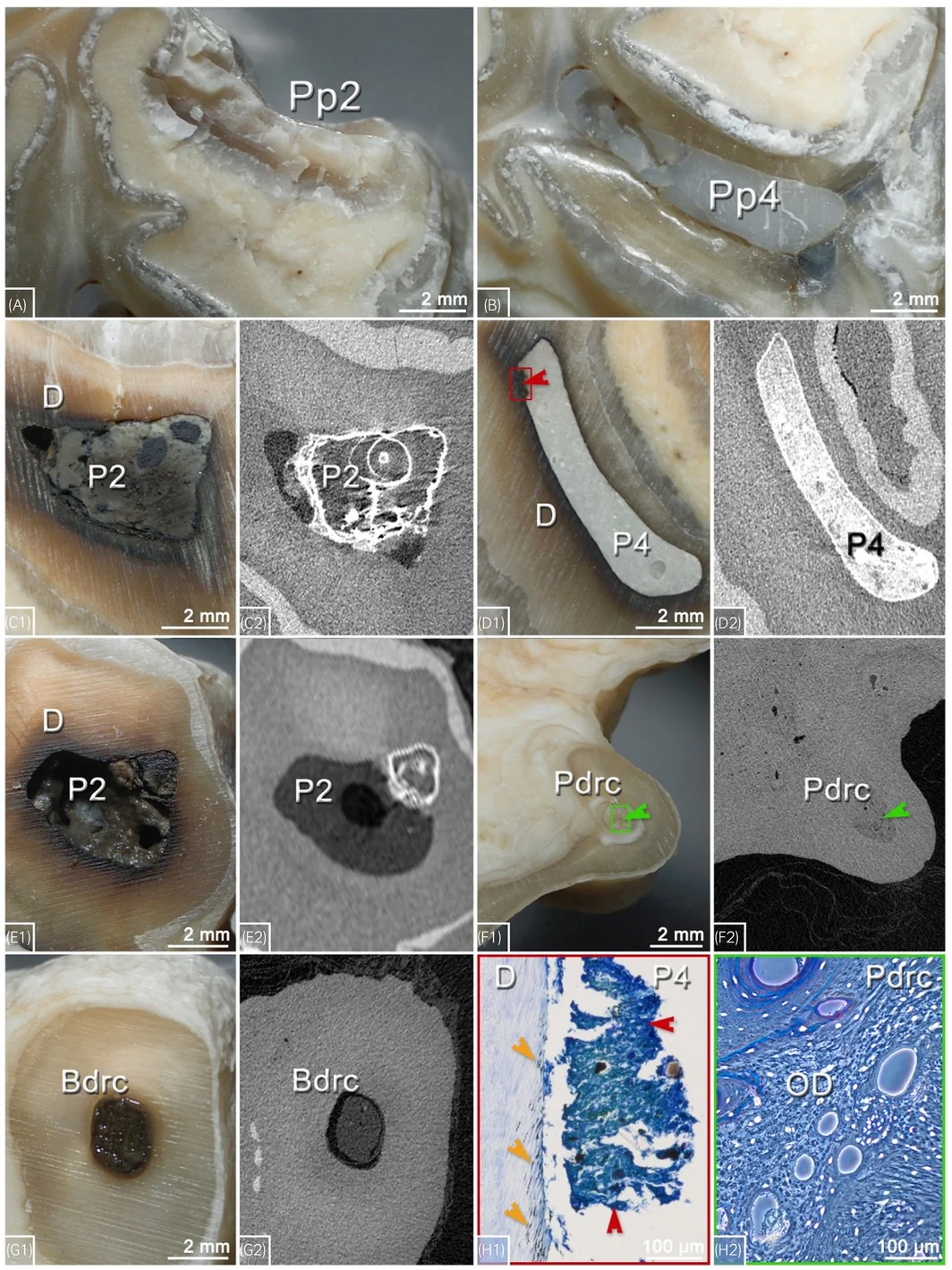
Stereomicroscopic, μCT, and histological images of Case 6 documenting the effect of eCSC in Triadan 207 extracted 2 years after treatment. (A) Occlusal surface, fracture involving pulp position 2 (Pp2). (B) Occlusal surface, pulp position 4 (Pp4) is filled with resin composite. Images C1/C2, E1/E2, G1/G2 document pulp compartments continuous with pulp position 2. (C1) Horizontal section 10 mm subocclusally, pulp horn 2 (P2) contains necrotic organic tissue mixed with eCSC. The peripulpal dentine appears discoloured black. (C2) Corresponding μCT image, the hyperattenuating (white) lines indicate the presence of eCSCs in the necrotic, organic tissue. (E1) Horizontal section 25 mm subocclusally, pulp horn 2 (P2) is partly filled with necrotic organic tissue. The peripulpal dentine appears discoloured black. (E2) Corresponding μCT image, the hyperattenuating eCSC is found only in a small portion of the pulp horn. (G1) Horizontal section through the buccodistal root canal (Bdrc), which is filled with necrotic organic tissue. (G2) Corresponding μCT image, no eCSC is present in this area. Images D1/D2, F1/F2, H1/H2 document pulp compartments continuous with pulp position 4. (D1) Horizontal section 10 mm subocclusally, pulp horn 4 (P4) is almost completely filled with eCSC, except for a small area containing necrotic organic material (red arrowhead). The red rectangle refers to histological image h1. (D2) Corresponding μCT image confirming that pulp horn 4 is almost completely filled with the hyperattenuating eCSC. (F1) Horizontal section through the palatodistal root canal (Pdrc), which contains a mixture of mineralised and non‐mineralised tissue components (green arrowhead). The green rectangle refers to histological image H2. (F2) Corresponding μCT image, confirming the presence of porous mineralised tissue within the palatodistal root canal. (H1) Histological image (toluidine blue stain) taken from the marked region (red rectangle) in D1. A mixture of necrotic tissue, eCSCs, and plant debris is visible (red arrowheads). In the adjacent peripulpal dentine (D) some dentinal tubules show the presence of dark‐stained particles (orange arrowheads), suggesting remnants of filling material. (H2) Histological image (toluidine blue stain) taken from the marked region (green rectangle) in F1. The palatodistal root canal (Pdrc) is filled with porous osteodentin (OD).

In the successful case (Case 7), there was adequate obturation of the pulp horns and sufficient apexification of the associated root canals. Examination of the periapical region, periodontal ligament, and surrounding alveolar bone showed no evidence of active infection or inflammatory infiltrate (Figure 4). The periodontal ligament appeared intact and of normal morphology. No adverse tissue reaction to eCSC was identified in the periapical tissues.

**FIGURE 4:**
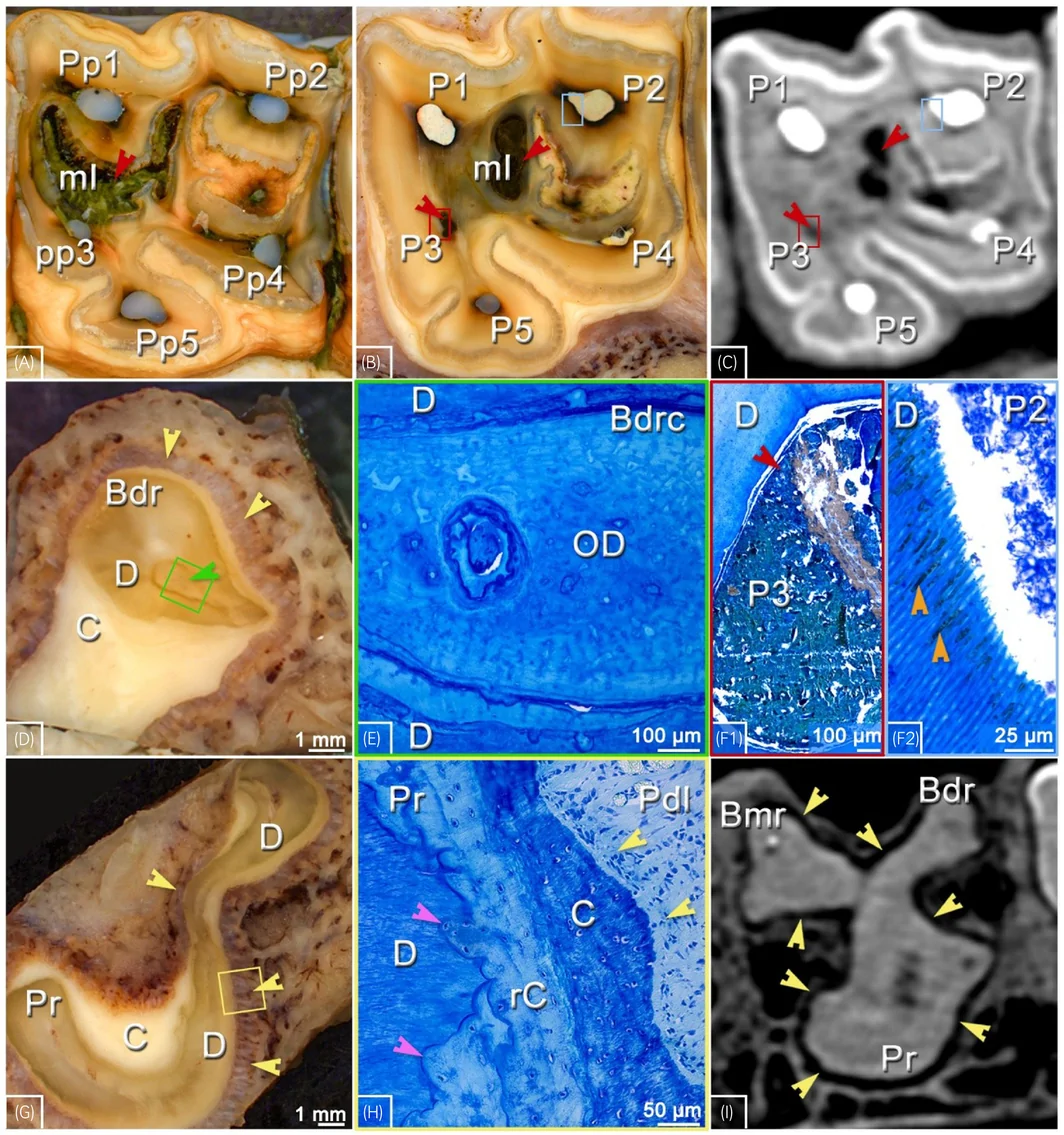
Stereomicroscopic, CT, and histological images of Case 7 documenting the effect of eCSC in a post mortem 209 submitted in situ with surrounding alveolus 16 months after treatment. (A) Occlusal surface, all pulp positions (Pp1–Pp5) are filled with resin composite. The mesial infundibulum (mI) showed marked cement hypoplasia and was filled with food debris (red arrowhead). (B) Horizontal section 25 mm subocclusally, pulp horns P1, P2 and P4 contain eCSCs, P5 shows presence of resin composite. Pulp horn 3 (P3) and the mesial Infundibulum (mI) contain necrotic organic tissue (red arrowheads). The red and blue rectangles refer to histological image shown in F1 and F2. (C) CT image corresponding to B. (D) Horizontal section through the buccodistal root (Bdr). The buccodistal root canal is filled with a mineralised tissue (green arrowhead). The periodontal space is filled with connective tissue and shows no signs of pathological changes (yellow arrowheads). The green rectangle refers to histological image shown in E. D = Dentine, C = Cementum. (E) Histological image (toluidine blue stain) taken from the marked region (green rectangle) in D. The buccodistal root canal (Bdrc) is filled with osteodentin (OD). D = Dentine. (F1) Histological image (toluidine blue stain) taken from the marked region (red rectangle) in C. A mixture of necrotic tissue, eCSCs, and plant debris (red arrowheads) is visible in Pulp horn 3 (P3). (F2) Histological image (toluidine blue stain) taken from the marked region (blue rectangle) in C. eCSCs is visible in Pulp horn 2 (P2). In the adjacent peripulpal dentine (D) some dentinal tubules show the presence of dark‐stained particles (orange arrowheads), suggesting remnants of filling material. (G) Horizontal section through the palatal root (Pr). The periodontal space is filled with connective tissue and shows no signs of pathological changes (yellow arrowheads). The yellow rectangle refers to histological image shown in H. D = Dentine, C = Cementum. (H) Histological image (toluidine blue stain) taken from the marked region (yellow rectangle) in G. The dentine (D) shows signs of a previous external root resorption in the form of lacuna‐like reversal lines (pink arrowheads). The earlier resorption lacunae have been filled with reparative cement (rC). Layers of regular extrinsic fibre cementum (C) and a regular periodontal attachment (yellow arrowheads) are visible at the periphery. Pdl = Periodontal ligament. (I) CT‐image of the root area displaying the buccomesial root (Bmr), buccodistal root (Bdr) and the palatal root (PR) interconnected by interradicular cementum. The apical aspect of the tooth is surrounded by a delicate, regular alveolar bone and a corresponding regular periodontal space (yellow arrowheads).

## 4 DISCUSSION

This case series reports the first clinical outcomes of orthograde equine endodontic treatment using a novel flowable eCSC. In cheek teeth, there was a success rate of 6 out of 7 cases, with 16 months to 5 years 4 months of follow‐up. These findings are consistent with the upper range of success rates reported for equine cheek tooth endodontic treatment.^3^, ^9^, ^10^ Additionally, this study provides preliminary clinical evidence supporting the translational relevance of eCSC from the laboratory to the clinic.

The principal advantage of CSCs over CHP as an obturation material relates to CSCs' lower solubility, good seal, antibacterial activity and bacterial entombment, non‐cytotoxicity and capacity for dentinal tubule penetration and intratubular mineralisation.^17^, ^20^, ^21^, ^30^, ^34^ CSCs have been shown to induce reparative dentinogenesis, leading to hard tissue repair by newly differentiated odontoblast‐like cells, and stimulate hard‐tissue‐forming cells to form matrices and then mineralisation of the tissues. This also leads to a better sealing ability and structural stability compared to CHP.^23^ In human endodontics, the use of CHP as a permanent obturation material is associated with halved periapical healing success rates compared to insoluble core materials such as gutta‐percha.^14^

As equine cheek teeth undergo continuous occlusal attrition,^35^ progressive exposure of the obturation material to the oral environment is inevitable, making the lower solubility of CSC particularly advantageous. While the restorative layer does require replacing every few years, this is a relatively simple process as the canals are still well sealed with the eCSC. In comparison, the primary author's experience using CHP as the obturation material has been that even with an interim layer of calcium hydroxide cement (Provicol) between the restoration and the CHP, feed ingress is commonly found before the restorative layer can be repeated. This necessitates complete endodontic retreatment of the tooth.

The single failed eCSC case (Case 6) in this series was attributable to incomplete canal debridement, as confirmed with histological evaluation. This finding highlights the enduring challenge of thorough biomechanical preparation in the complex, multi‐canaled and multi‐rooted anatomy of equine cheek teeth.^27^, ^28^, ^29^ The complex pulp morphology, as well as limited space and visibility, makes complete mechanical debridement, even with dedicated equine endodontic instrumentation, challenging.^27^ Notably, eCSC was not implicated as the cause of failure in this case, and no adverse reaction to the material was identified histopathologically in either the failed or successful case examined. This is consistent with the well‐established biocompatibility of Portland cement‐based CSCs in both human and veterinary contexts.^36^, ^37^, ^38^, ^39^, ^40^, ^41^ Interestingly, concern also developed regarding the immune capabilities of this horse after the endodontic treatment, and the horse was subsequently diagnosed with PPID. As such, a degree of immunocompromise may have contributed to the treatment failure. While every effort is made to thoroughly clean and disinfect the pulp canals, due to the complex anatomy, complete sterilisation is not a realistic goal, so success relies on the horse's immune system to assist in containing and destroying any residual infection. Therefore, caution is advised in treating horses with reduced immune capabilities, such as those with PPID or systemic illness.

One eCSC case (Case 3) sustained a buccal slab fracture 18 months after the endodontic procedure. It is considered that this was likely due to increased tooth weakening secondary to all five pulp cavities being endodontically affected prior to treatment. At the initial treatment a wide, dark fissure fracture was identified running through pulp canal 2 into the distobuccal corner, and another running from the mesial aspect of the tooth to pulp canal 1, and it may be possible that if left untreated, the tooth may have fractured earlier/further without the added strength bestowed by the eCSC. Extraction was discussed with the owner following fracture identification, noting that any further fracture development would make a subsequent extraction more difficult. However, given the tooth appeared apically quiescent, the owner elected to leave the tooth in situ and continue monitoring rather than extract the tooth. As the exposed obturation material was set hard within the tooth, only replacement of the occlusal restoration was required. This was advantageous compared with CHP, which would have required a full retreatment due to contamination and feed ingress. No further progression has been observed in the subsequent 3 years. The presence of the concrete‐like eCSC material within the pulp cavities is likely to have been additionally advantageous following fracture as it adds strength to the tooth and may make the tooth more resistant to subsequent fracture.^26^

The CT and histopathological data from one of the successful cases in this series provide two complementary forms of evidence for biological healing. The CT demonstrated healing of a small fistulous sinus tract following eCSC obturation, representing a clinically meaningful resolution that is directly relevant to equine practitioners. The histopathological examination provided tissue‐level confirmation that the periodontal tissues are free of inflammatory and infectious processes and that there is no foreign body reaction to the eCSC material. Furthermore, the histologically visible, sequential arrangement of formations consisting of resorption lacunae, reparative cementum, and regular extrinsic fibre cementum suggests an induction of remodelling processes to restore a functional periapical apparatus consisting of the periapical cementum, the periodontal ligament, and the alveolar bone after eCSC treatment. Particularly in horses, whose teeth undergo a continuous process of eruption, it is crucial to ensure not only a periodontal environment free of inflammation and pain, but also one that is functionally sound. Together, these findings support the interpretation that eCSC can function as a biologically compatible, clinically effective obturation material in equine cheek teeth.

Although there are many positive aspects to the use of CSCs, hard‐setting after curing represents a practical difficulty of CSCs, as it renders retreatment or revision difficult in non‐healing cases^42^ and may increase the complexity of extraction if required. Conversely, CHP is relatively easily removed from the canal and allows revision of treatment in refractory cases.^43^ Endodontically treated teeth have been previously recognised to have reduced fracture resistance,^44^ and this should be discussed with owners as a long‐term consideration, noting that a failed endodontically treated tooth may be more challenging to extract than a previously untreated tooth. Treatment with CSC's, however, has been shown to increase fracture resistance in human studies compared to other treatments^26^ and may help reduce this risk compared to traditional treatments such as CHP.

Several limitations of the present study must be acknowledged. The case numbers are small and there was also heterogeneity in follow‐up duration, imaging modality, and assessment methodology. All procedures were performed by a single experienced operator and outcomes in less experienced hands could differ. CT imaging, which provides superior spatial resolution for periapical assessment compared to radiography, was available in only one case, with earlier cases reliant solely on two‐dimensional radiography and clinical examination, which may underestimate the extent of disease and/or periapical healing. Additionally, no standardised success criteria specifically validated for equine cheek tooth endodontics currently exist in the literature to the author's knowledge, and the criteria employed in the present study were adapted from those used in human and equine incisor endodontic research.^32^, ^33^

Despite these limitations, the data presented here support the case for a prospective clinical trial evaluating eCSC for equine cheek tooth endodontics, with standardised case selection criteria, occlusal filling depth, validated success/failure definitions, uniform follow‐up intervals, and inclusion of CT imaging as a primary outcome measure where feasible. Such a trial would provide the higher‐quality evidence base needed to define the indications, contraindications, and predictability of eCSC for clinical use.

## 5 CONCLUSIONS

This case series demonstrates that orthograde endodontic treatment of equine teeth using a flowable CSC (eCSC) can produce durable clinical success. CT imaging confirmed resolution of an odontogenic sinus fistula in one case, and histopathological examination revealed periapical healing with no evidence of adverse tissue reaction to eCSC. The sole failure with eCSC in the series was attributed to incomplete canal preparation and potentially reduced patient immune system capabilities rather than a material‐specific complication. These preliminary clinical findings support the continued development and investigation of eCSC as an alternative to CHP for equine cheek tooth endodontic obturation.

## FUNDING INFORMATION

A research grant from the Murdoch University Vet Fund funded the repeat CT examination. Dr. Erin Kelty is funded by a Stan Perron Charitable Foundation Fellowship and was previously supported by a National Health and Medical Research Council Fellowship.

## CONFLICT OF INTEREST STATEMENT

The authors declare no conflicts of interest.

## AUTHOR CONTRIBUTIONS

**Kirsten Jackson:** Conceptualization; investigation; writing – original draft; writing – review and editing; project administration; methodology; data curation. **Josephine Faulkner:** Funding acquisition; writing – review and editing; formal analysis; data curation; validation; investigation; visualization; software. **Carsten Staszyk:** Investigation; validation; visualization; writing – review and editing; formal analysis; data curation; software. **William Ha:** Conceptualization; investigation; writing – review and editing; supervision; methodology. **Ebony Quintrell:** Writing – review and editing; data curation; supervision. **Erin Kelty:** Conceptualization; investigation; funding acquisition; writing – review and editing; project administration; data curation; supervision; methodology.

## DATA INTEGRITY STATEMENT

Kirsten Jackson had full access to all the data in the study and takes responsibility for the integrity of the data and the accuracy of data analysis.

## ETHICAL ANIMAL RESEARCH

Research ethics committee oversight not required by this journal: retrospective study of clinical records.

## INFORMED CONSENT

At the time of treatment, signed, informed owner consent was obtained for all procedures.

## Supporting information


**Table S1.** Summary of cases treated with equine Calcium Silicate Cement (eCSC).

## Data Availability

The data that support the findings of this study are available upon reasonable request from the corresponding author. Open data sharing exemption granted by the editor.
