# Supplementary material for: Clinical outcomes of a novel flowable calcium silicate cement in equine endodontic treatment
Source: Equine Vet J. 2026 Aug 5;58(6):1602–11. doi: 10.1002/evj.70299 (PMC13657192; doi:10.1002/evj.70299)
Supplement: Supplementary file 1 [file EVJ-58-1602-s001.docx]

| **Case** | **Signalment** | **Affected tooth (Triadan) and presenting signs** | **Number of stages in treatment** | **Follow-up duration (number of follow up appointments)** | **Outcome** | **Findings at follow up appointments** | **Diagnostics and post procedure monitoring performed** |
| --- | --- | --- | --- | --- | --- | --- | --- |
| 1 | 23yo Arabian Mare | 201  NVPE, apical periodontitis on radiographs | 1 | 3 years 11 months (5) | Success | Tooth still in place and no sclerosis radiographically. Possibly a cementoma apically. No external or radiographic signs of inflammation, no draining tracts on probing. | Pre and post procedure radiographs |
| 2 | 9yo Morgan Gelding | 207  Unilateral nasal discharge, NVPE P2, P4, apical periodontitis on radiographs | 2 | 5 years 4 months (5) | Success | Nasal discharge ceased within a few days of treatment and hasn’t returned, radiographic improvement | Pre and post procedure radiographs |
| 3 | 10yo WB Mare | 108  NVPE P1, P2, P3, P4, P5, apical periodontitis on radiographs | 2 | 4 years 6 months (8) | Success | Radiographic improvement. Sustained a buccal slab fracture through P1, P2 18 months post procedure but still sealed with eCSC so restoration replaced.  Replaced occlusal restoration on P3, P4, P5 at 2 years 10 months. No further fractures | Pre and post procedure radiographs |
| 4 | 17yo Friesian Mare | 207  NVPE P1, P3, P5, apical periodontitis on radiographs | 1 | 4 years 5 months (6) | Success | Radiographic improvement | Pre and post procedure radiographs |
| 5 | 15yo WB Gelding | 206  Quidding, ear droop on left side, NVPE P2, P4, apical periodontitis on radiographs | 1 | 3 years 4 months (4) | Success | Clinical signs resolved, radiographic improvement | Pre and post procedure radiographs |
| 6 | 16yo Anglo-Arabian Mare | 207  NVPE P2, P4, apical periodontitis on radiographs. | 2 | 2 years 3 months (5) | Failure | After 2 years 3 months, P1, P3, P5 became open occlusally, tooth was extracted | Pre and post procedure radiographs and histology on the extracted tooth |
| 7 | 15yo Irish Sport Horse Mare | 209  NVPE P1, P2, P3, P4, P5, periapical changes on radiographs and CT, and a draining tract to the sinus on CT | 2 | 1 year 4 months (2) | Success | Sinus infection never developed, healing of periapical infection and draining tract on radiographs and CT. Also histological resolution of infection and healthy periapical area. | Pre and post procedure radiographs and CT, post mortem histology |
